# Cellular metabolomics reveals the osteogenic mechanism of MC3T3-E1 cells in porous Ti6Al4V scaffolds under low-intensity pulsed ultrasound

**DOI:** 10.3389/fcell.2026.1945138

**Published:** 2026-09-24

**Authors:** Xinyun Li, Lin Wu

**Affiliations:** 1 Department of Stomatology, Affiliated Hospital of Guangdong Medical University, Zhanjiang, Guangdong, China; 2 Department of Stomatology, Shengjing Hospital of China Medical University, Shenyang, Liaoning, China

**Keywords:** cellular metabolomics, large-area bone defects, low-intensity pulsed ultrasound, osteogenic differentiation, porous titanium alloy scaffolds

## Abstract

**Introduction:**

Porous titanium alloy scaffolds offer multiple advantages in treating large-area bone defects; however, their poor bioactivity limits their application. Low-intensity pulsed ultrasound (LIPUS) serves as a non-invasive method to enhance bone regeneration in porous titanium-6aluminium-4vanadium (Ti6Al4V) alloy scaffolds. This study aimed to examine the biological behavior and metabolism of murine osteoblastic MC3T3-E1 cells in porous Ti6Al4V (pTi64) scaffolds under LIPUS.

**Methods:**

MC3T3-E1 cells were incubated in pTi64 scaffolds and stimulated with LIPUS. Cell morphology, viability, and osteogenic-related gene expression were evaluated *in vitro*. Intracellular metabolites of MC3T3-E1 cells incubated in pTi64 scaffolds and stimulated with LIPUS for 7 days were analyzed using an ultraperformance liquid chromatography coupled with a triple quadrupole-linear ion trap mass spectrometer (UPLC-QTRAP-MS)-based cellular metabolomics approach.

**Results:**

*In vitro*, MC3T3-E1 cells in the pTi64+LIPUS group exhibited enhanced morphology, viability, and osteogenic-related gene expression compared with the pTi64 group, suggesting that LIPUS enhances cell morphology, reduces apoptosis, and promotes osteogenic differentiation in pTi64 scaffolds. LIPUS treatment altered the cellular metabolic network; 86 differential metabolites were identified. Alterations in metabolic pathways were associated with the one-carbon pool by folate, pantothenate and coenzyme A biosynthesis, as well as the metabolism of amino acids, glycerolipids, and arachidonic acid.

**Discussion:**

These findings offer novel insights into the osteogenic mechanisms of MC3T3-E1 cells in pTi64 scaffolds under LIPUS.

## 1 Introduction

Repairing large-area bone defects resulting from tumours, infections, trauma, or congenital conditions remains a significant challenge (Zhu et al., 2023). Due to the limitations in bone tissue’s ability to repair large defects and the constraints of autologous and allogeneic bone transplantation, artificial bone replacement materials have garnered significant attention (Zhao et al., 2021). There has been a collaboration of tissue engineering with medicine to promote the development of techniques to repair bone damage and increase the quality of life of patients. Recently, three-dimensional (3D) printed titanium alloy scaffolds have been widely used in plastic and reconstructive surgery because they have excellent biocompatibility and mechanical properties and provide a suitable macrostructure to support bioremediation (Gao et al., 2020; Zhang et al., 2020; Gu et al., 2022). However, the poor bioactivity of titanium alloys limits the application of 3D scaffolds, and bone tissue may not grow in the centre of the scaffold (Wang et al., 2023). Therefore, it is necessary to develop innovative clinical methods to enhance nutrient delivery and accelerate cell proliferation, differentiation, and stabilisation inside porous structures.

Low-intensity pulsed ultrasound (LIPUS) is recognized as a non-invasive therapeutic method for treating bone fractures. LIPUS is transmitted into living tissues in the form of acoustic pressure waves, which can produce micromechanical strains in biological tissues to accelerate tissue healing (Dubernard et al., 2024; Zong et al., 2025). In recent years, it has been confirmed that LIPUS not only promotes the healing of fractures and bone non-union by directly regulating osteoblasts (Cao et al., 2025), osteoclasts (Meng et al., 2018), and mesenchymal stem cells (Costa et al., 2018), but also has a positive effect on the exchange of nutrients (Liu et al., 2019), angiogenesis (Wu et al., 2018) and neuromodulation (Hu et al., 2023). Therefore, we propose that LIPUS can serve as an innovative clinical approach to accelerate osteogenesis in 3D-printed porous titanium-alloy scaffolds. A preliminary experiment by our research group concluded that LIPUS accelerates bone ingrowth into porous titanium-6aluminium-4vanadium (Ti6Al4V) scaffolds *in vivo* (Qin et al., 2025). However, the osteogenic mechanisms of LIPUS on osteoblasts inside porous scaffolds are still unclear, and further experiments are needed to deepen our understanding. Understanding how LIPUS enhances osteogenesis in porous scaffolds is crucial for supporting the use of 3D scaffold materials combined with LIPUS in treating large-area bone defects.

Metabolomics, complementing genomics, transcriptomics, and proteomics, is an effective approach for quantifying global or targeted endogenous metabolites to elucidate metabolic processes through the analysis of biological specimens (Johnson et al., 2016). Understanding how endogenous metabolites respond to intrinsic and extrinsic factors is crucial for elucidating the functions of cells, biofluids, tissues, and organisms and their environment interactions (Lin et al., 2024). A metabonomic approach offers benefits for comprehending bone cell behaviour, diagnosing, and prognosing of diseases, and developing treatments like biomaterial implants, thereby advancing bone research (Fan et al., 2021). Metabolomics, the extensive analysis of the metabolites involved in biochemical processes, is anticipated to enhance our understanding of LIPUS’s role in promoting osteogenesis within porous scaffolds. Describing the changing metabolite pathways during LIPUS stimulation can greatly improve the understanding of the osteogenesis mechanism promoted by LIPUS. Despite numerous studies utilizing liquid chromatography-mass spectrometry (He et al., 2020; Lv et al., 2020), gas chromatography-mass spectrometry (Batushansky et al., 2019), and nuclear magnetic resonance (Liang et al., 2020; Liu et al., 2020) to investigate metabolic changes in bone diseases, the cellular metabolomics of osteoblasts in porous Ti6Al4V (pTi64) scaffolds under LIPUS remains underexplored. We hypothesized that LIPUS promotes osteogenic differentiation of osteoblasts in pTi64 scaffolds through the regulation of intracellular metabolism. To test this hypothesis, we examined the metabolic alterations in MC3T3-E1 cells following LIPUS stimulation using a widely targeted metabolomics approach.

This study examined the biological behaviour of murine osteoblastic MC3T3-E1 cells in pTi64 scaffolds subjected to LIPUS. We utilized global metabolomics via ultra-performance liquid chromatography coupled with a triple quadrupole-linear ion trap mass spectrometer (UHPLC-QTRAP-MS/MS) combined with multivariate statistical data analysis to investigate alterations in metabolic signatures and pathways in these osteoblasts.

## 2 Materials and methods

### 2.1 Preparation of the pTi64 scaffolds

A porous scaffold exhibiting an interconnected network and an extra-low interstitial content interconnected was fabricated using an electron beam melting (EBM) system (Arcam AB, Sweden). The monolithic parts were built layer-by-layer from medical-grade Ti6Al4V alloy. Arcam AB supplied the medical-grade Ti6Al4V precursor powders, which had an average particle diameter of approximately 50 μm. The EBM system operated under a vacuum of 10–3 mbar, utilizing a 60 kV voltage and an electron beam approximately 200 μm in size. Fabrication commenced with preheating of the powder prior to melting. Each layer underwent a pre-scan, followed by 11scans of the powder layer at a velocity of 15,000 mm/s and a beam current of 30 mA. The powder layer was heated to ∼730 °C. After preheating, the melting of selected areas was controlled using a computer-aided design program. The Young’s modulus and compressive strength of the studied pTi64 scaffolds are 10 GPa and 150 MPa, respectively. The pTi64 scaffolds contained fully interconnected pores with average diameter of 400–500 μm and were prepared with a height of 3 mm and diameter of 10 mm, with 65%–70% porosity. These microstructures allow good osteoconductivity. The samples were cleaned through ultrasonic agitation and sterilised in an autoclave at 134 °C/0.21 MPa before the experiment.

### 2.2 Cell culture and seeding

The MC3T3-E1 osteoblasts (passage-16 cells) were provided by Central Laboratory, School of Stomatology, China Medical University. The cells were cultured in a basal medium of alpha-modified minimum essential medium (α-MEM, HyClone, USA) with 10% fetal bovine serum (FBS, Clark, Australia) at 37 °C, 95% humidity, and 5% CO_2_. The culture medium was refreshed every 3 days, and cells were subcultured at 80% confluence in a 1:3 ratio.

Prior to cell seeding, the scaffolds were pre-wetted in culture medium for 4–6 h using vacuum equipment. Four scaffolds were placed in the centre of each well of 6-well plates. In all subsequent formal experiments, a 20 μL cell suspension containing 5 × 10^4^ cells was applied to each scaffold in 6-well plates and incubated for 2 h. Subsequently, 3 mL of medium was added to each well, and the scaffolds were incubated for 24 h to ensure firm cell attachment. The remaining samples were randomly allocated into two groups: one receiving LIPUS stimulation (pTi64+LIPUS group) and the other receiving sham stimulation (pTi64 group), with incubation periods of 4, 7, or 14 days. The medium was replaced every 3 days. For each experiment, a minimum of three independent biological replicates (n = 3) were conducted, with each replicate derived from a distinct culture well containing four scaffolds.

### 2.3 LIPUS stimulation

A Sonicator 740 (Mettler Electronics, Anaheim, CA, USA) equipped with a 10 cm^2^ transducer was employed to deliver the ultrasound stimulation. Cells cultured in porous scaffolds were positioned at the center of 6-well plates and subjected to daily stimulation for 20 min throughout the experiment. The stimulation parameters were set at 30 mW/cm^2^, 1 MHz frequency with a 200 μs pulse duration repeated at 100 Hz (Uddin and Qin, 2013; Uddin et al., 2013; Sun et al., 2020). The ultrasonic transducer was positioned 5 cm above the bottom of the 6-well plates. To reduce the attenuation of acoustic intensity during propagation, both the transducer and the plates were submerged in degassed water (Casarotto et al., 2004; Poltawski and Watson, 2007). The sham control group was subjected to the identical experimental procedure, except that the ultrasound generator remained switched off.

### 2.4 Cell morphology and ingrowth depth assay

Cells were seeded on samples and subjected to either LIPUS or sham stimulation for 4 days. Subsequently, the samples were fixed with 4% paraformaldehyde in PBS for 20 min and permeabilized with 1% Triton-X 100 in PBS for 25 min. Following three PBS washes, samples were stained with fluorescein isothiocyanate (FITC)-phalloidin (Abcam, Cambridge, MA, USA) for 60 min at 23 °C–25 °C to visualize filamentous actin (F-actin), with nuclei counterstained using 4′,6 diamidino-2-phenylindole (DAPI). Images were captured using an Olympus confocal laser scanning microscope. The confocal laser microscope scanned the scaffolds in layers. The scaffolds were scanned every 10 μm and 85 images were captured. Images were sequentially layered to construct a three-dimensional structure of MC3T3-E1 cells in the pTi64 scaffolds. Due to the hardware constraints of the z-axis platform and the working distance limitations of the objective lens, the maximum achievable scanning depth of the confocal system was 850 μm. Consequently, all three-dimensional reconstructions and morphological analyses reflect the characteristics of the cell population within the 850 μm region of the scaffold’s top layer. Image-Pro Plus software was utilized to measure the ratio of the nuclear area to the cell overall area.

### 2.5 LIVE/DEAD cell fluorescent stain and apoptosis

When cells in the different groups were cultured for 4 days, a live-and-dead cell assay (Abcam, Cambridge, MA, USA) was used to visualise the live and dead cells. Samples were stained with 1× live/dead dye for 10 min at 23 °C–25 °C and observed under a fluorescence microscope using a ×10 objective. Live cells were labeled with green with FITC (green fluorescent dye) while dead cells were labeled with red Texas Red (red fluorescent dye). The dead/total cell ratio (DC/TC) was calculated by analyzing six randomly chosen microscopic fields per sample.

Following a 4 days incubation period for the various groups, cell apoptosis rates were assessed via Annexin V-FITC/propidium iodide (PI) double-staining and flow cytometry (FACS Vantage SE, BD Biosciences).

### 2.6 Detection of alkaline phosphatase (ALP) and quantitative real-time PCR (qPCR)

ALP activity in each sample was assessed at 4 and 7 days post-LIPUS or sham stimulation using p-nitrophenyl phosphate (Sigma, Ventura, USA) and a spectrophotometric plate reader set to 405 nm. Protein concentration was measured with a micro-bicinchoninic acid assay kit. ALP activity was standardized to total protein concentration and reported as enzyme activity units per Gram of protein.

RNAiso Plus (Takara, Shiga, Japan) was used to extract total RNA from cells subjected to either LIPUS or sham stimulation for 7 days. RNA concentration was assessed with a NanoDrop 2000 spectrophotometer (Thermo Fisher Scientific, Wilmington, DE, USA). Complementary DNA was synthesised using the PrimerScript RT Reagent Kit (Takara, Shiga, Japan). qPCR was conducted using TB Green Premix Ex Taq II (Takara, Shiga, Japan) on the Bio-Rad CFX96 PCR Manager system (Applied Biosystems, Foster City, CA, US). qPCR was conducted to assess the expression levels of *Alp* (forward: GCA​GTA​TGA​ATT​GAA​TCG​GAA​CAA​C, reverse: ATG​GCC​TGG​TCC​ATC​TCC​AC), runt-related transcription factor-2 (*Runx2*, forward: CGG​CAA​GAT​GAG​CGA​CGT​GAG, reverse: TGC​TGC​TGC​TGC​TGC​TGT​TG), collagen type 1 (*Col-1*, forward: GCA​TGG​CCA​AGA​AGA​CAT​CC, reverse: CCT​CGG​GTT​TCC​ACG​TCT​C), and *Ocn* (forward: CTG​GCT​GCG​CTC​TGT​CTC​T, reverse: TGC​TTG​GAC​ATG​AAG​GCT​TTG). All data were normalised to *Gapdh* (forward: AAG​AAG​GTG​GTG​AAG​CAG​GCA​TC, reverse: CGG​CAT​CGA​AGG​TGG​AAG​AGT​G).

### 2.7 OCN immunofluorescence staining and imaging

Cells seeded on samples treated with LIPUS or sham stimulation for 7 and 14 days were fixed in 4% paraformaldehyde for 20 min, permeabilized with 1% Triton X-100 for 10 min, and blocked with 5% bovine serum albumin for 30 min at room temperature. Cells were stained overnight at 4 °C with a 1:200 dilution of OCN rabbit polyclonal antibody (Proteintech, USA) in 3% bovine serum albumin in PBS, followed by 1-h incubation at room temperature with goat anti-rabbit IgG-rhodamine (Bioss Biotechnology, Beijing, China) at the same dilution.

Following OCN staining, samples were treated with FITC-phalloidin (Abcam, Cambridge, MA, USA) for 30 min at 23 °C–25 °C to visualize F-actin, counterstained with DAPI, and examined using a Zeiss Spot fluorescence microscope (Carl Zeiss MicroImaging GmbH, Göttingen, Germany). Images were obtained with a Zeiss LSM 710 confocal microscope at ×10 magnification. Image-Pro Plus software was utilized to measure the average fluorescence intensity of OCN from five randomly selected fields per sample.

### 2.8 Metabolomics

#### 2.8.1 Sample preparation and metabolites extraction

After seeding cells on samples subjected to either LIPUS or sham stimulation for 7 days, the samples were washed twice with pre-cooled PBS and treated with trypsin-EDTA for 5 min. Cell pellets were rapidly centrifuged and transferred into 2 mL EP tubes at a density of approximately 1 × 10^7^ cells per tube. Metabolites were extracted using 1.0 mL of a pre-cooled 80% methanol aqueous solution and vortexed for 2 min. The mixture underwent three cycles of freezing in liquid nitrogen for 3 min, followed by 5 min on ice and a 2 min of vortexing. The mixture was centrifuged at 1,200 rpm and 4 °C for 5 min. An equal volume of supernatants (200 μL) was transferred to ultra-performance liquid chromatography-tandem mass spectrometry vials for analysis. Quality control (QC) samples were created by combining 20 μL of supernatant from each sample for analysis. An analytical batch was used to evaluate system stability by inserting ten samples. The metabolomics analysis was conducted with six independent biological replicates per group.

#### 2.8.2 UPLC-QTRAP-MS/MS analysis

The metabolites were separated using UPLC coupled with a tandem mass spectrometry system (UPLC, ExionLC AD; MS, QTRAP® System). The UPLC column used was a Waters ACQUITY UPLC HSS T3 C18 with dimensions of 1.8 μm particle size and 2.1 mm × 100 mm. The chromatographic separation used a mobile phase comprising (A) deionised water with 0.1% formic acid for positive ion mode and 5 mmol/L ammonium acetate for negative ion mode, and (B) acetonitrile. The elution gradient was programmed as follows: A/B, 95/5 (V/V) at 0 min; transitioning to A/B, 10/90 (V/V) by 10 min, maintained until 11 min; then reverted to A/B, 95/5 (V/V) at 11.1 min and held constant until 14 min. The flow rate was set at 0.5 mL/min. The injection volume was 2 μL. The column temperature was kept at 40 °C. LIT and triple quadrupole (QQQ) scans were conducted using a QTRAP® LC-MS/MS System, a QTRAP with an ESI Turbo Ion-Spray interface, capable of operating in both positive and negative ion modes, and managed by Analyst 1.6.3 software (Sciex). The ESI source was operated with a source temperature of 500 °C and an ion spray voltage of 5500 V for positive mode and −4500 V for negative mode. The ion source gases I and II were set to 55 and 60 psi, respectively, while the curtain gas was maintained at 25.0 psi. The collision gas was set to a high level. Instrument tuning and mass calibration utilized 10 μmol/L polypropylene glycol solutions and in the QQQ mode and 100 μmol/L in LIT modes. Multiple reaction monitoring (MRM) transitions were monitored for each period based on the eluted metabolites.

#### 2.8.3 Data processing and analysis

Metabolite qualitative analysis utilized the Metware database, relying on retention time (RT), ion pair data, and secondary spectrum information. Metabolites were quantified using triple quadrupole mass spectrometry with MRM. The original data separation was assessed through unsupervised principal component analysis (PCA). Supervised orthogonal partial least square discriminant analysis (OPLS-DA) was performed to maximize covariance between the predictors and class membership in both positive and negative ionization modes. After Pareto scaling, the variable importance in projection (VIP) for each term in the OPLS-DA model was derived, and reliability was assessed using 7-fold cross-validation alongside 200 permutation tests. Variables with a VIP > 1 were further analysed using independent sample two-tailed Student's *t*-tests to evaluate the significance of each variable. Fold change (FC) was determined by comparing the mean relative peak intensities between the pTi64+LIPUS and pTi64 groups. Statistical analysis of the normalized values to identify significant metabolic changes between the pTi64+LIPUS and pTi64 groups was conducted using SPSS 22.0 software (SPSS Inc., Chicago, IL, USA). Differential metabolites were identified as variables with a VIP greater than 1.0, a FC less than 1.2 or below 0.83, and a P-value under 0.05. Data were expressed as mean ± SEM and visualized using GraphPad Prism 8. Hierarchical cluster analysis (HCA) of samples and metabolites was visualized as heatmaps, generated using the R package Complex Heatmap. In HCA, metabolite signal intensities, normalized by unit variance scaling were represented using a colour spectrum. The KEGG database (http://www.genome.jp/kegg) was utilized to enhance the understanding of metabolic pathways and construct a metabolomics regulation network.

## 3 Results

### 3.1 Cell morphology and scaffolds colonization

Cell surface interactions and cell adhesion involve complex processes including the reorganization of cytoskeletal proteins like actin, which are the early indicators of the regeneration. Therefore, we stained actin filaments with FITC-phalloidin and reconstructed three-dimensional images of cells in the Ti64 scaffold to observe cell morphology and colonisation. These images (Figures 1A,C) demonstrate that the cells completely spread on the scaffolds. Some cellular extensions stretched towards the pores of the Ti64 scaffolds were visible in the pTi64+LIPUS group; their morphology was better than that in the pTi64 group at 4 days. Statistical analysis revealed that the cellular to nuclear area ratios were significantly higher in the pTi64+LIPUS group compared to the pTi64 group (Figure 1B). These results suggest that LIPUS improves cell morphology and colonisation in pTi64 scaffolds.

**FIGURE 1:**
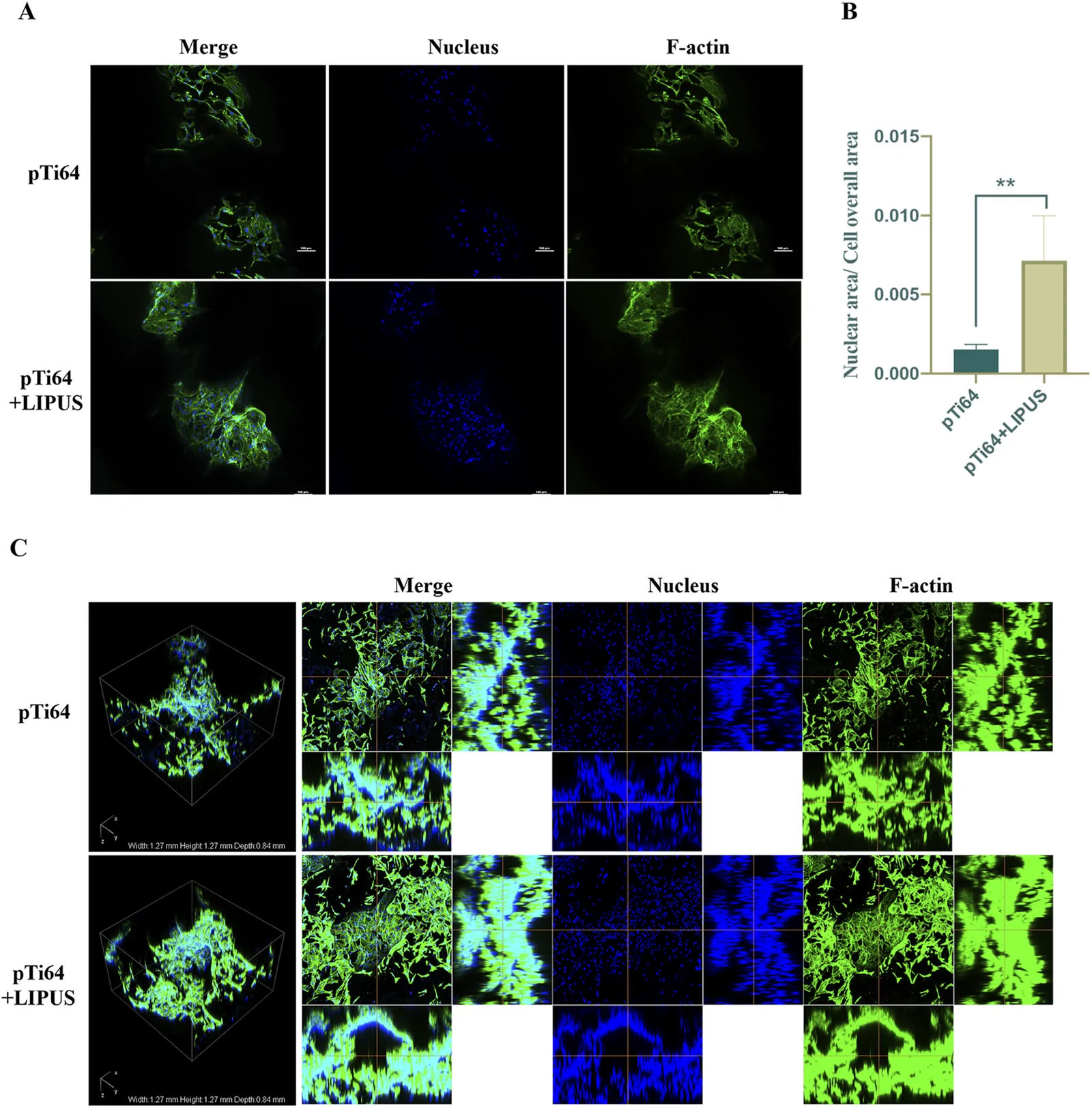
**(A)** Fluorescent staining of MC3T3-E1 cells in pTi64 group and pTi64+LIPUS group and **(B)** analysis of morphology (n = 3, **p < 0.01). Scale bar: 100 μm. **(C)** Three-dimensional images of cells (0–850 μm from the scaffold surface) in different groups (green, FITC-phalloidin for F-actin; blue, DAPI for nucleus).

### 3.2 Cell viability

Cell viability under various conditions was assessed using live/dead fluorescent staining. As shown in Figures 2A,B, demonstrate that the DC/TC ratio in the pTi64+LIPUS group was significantly reduced compared to the pTi64 group at 4 days.

**FIGURE 2:**
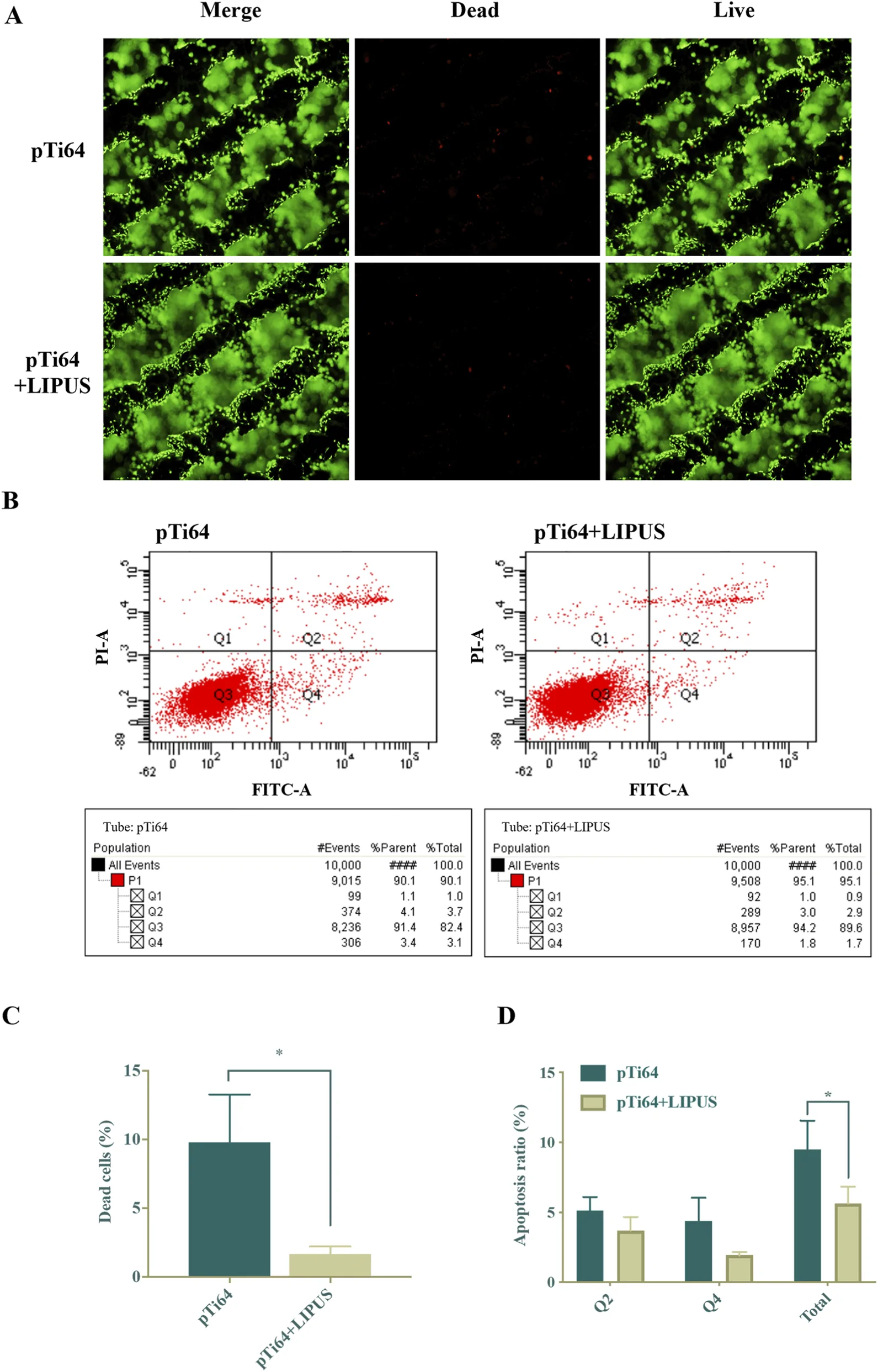
**(A)** Fluorescent staining images of live/dead cells after incubation for 4 days in different groups. Scale bar: 200 μm. **(B)** Typical images of cell apoptosis detected with Annexin V-FITC/PI double-staining using flow cytometer. Statistical analysis of dead cells percentage **(C)** and apoptotic ratio **(D)** in all the groups (n = 3, *p < 0.05).

Figure 2C illustrates the percentage of apoptotic cells. Statistical analysis indicated that variations in the apoptotic proportion mirrored those in the DC/TC percentage (Figure 2D). On day 4, the pTi64+LIPUS group exhibited a significantly lower proportion of apoptotic cells compared to the pTi64 group. Fluorescent staining and apoptosis assays demonstrated that LIPUS reduced apoptosis in MC3T3-E1 cells in pTi64 scaffolds.

### 3.3 Osteogenic response

#### 3.3.1 Analysis of ALP activity and osteogenic gene markers

Elevated ALP levels are thought to raise inorganic phosphate concentrations, a key element of bone mineral apatite. ALP activity, an early marker of osteoblast differentiation and maturation, was assessed after 4 and 7 days of incubation in pTi64 scaffolds, both with and without LIPUS stimulation. The ALP activity illustrated in Figure 3A was statistically higher in the pTi64+LIPUS group than in the pTi64 group at 4 and 7 days.

**FIGURE 3:**
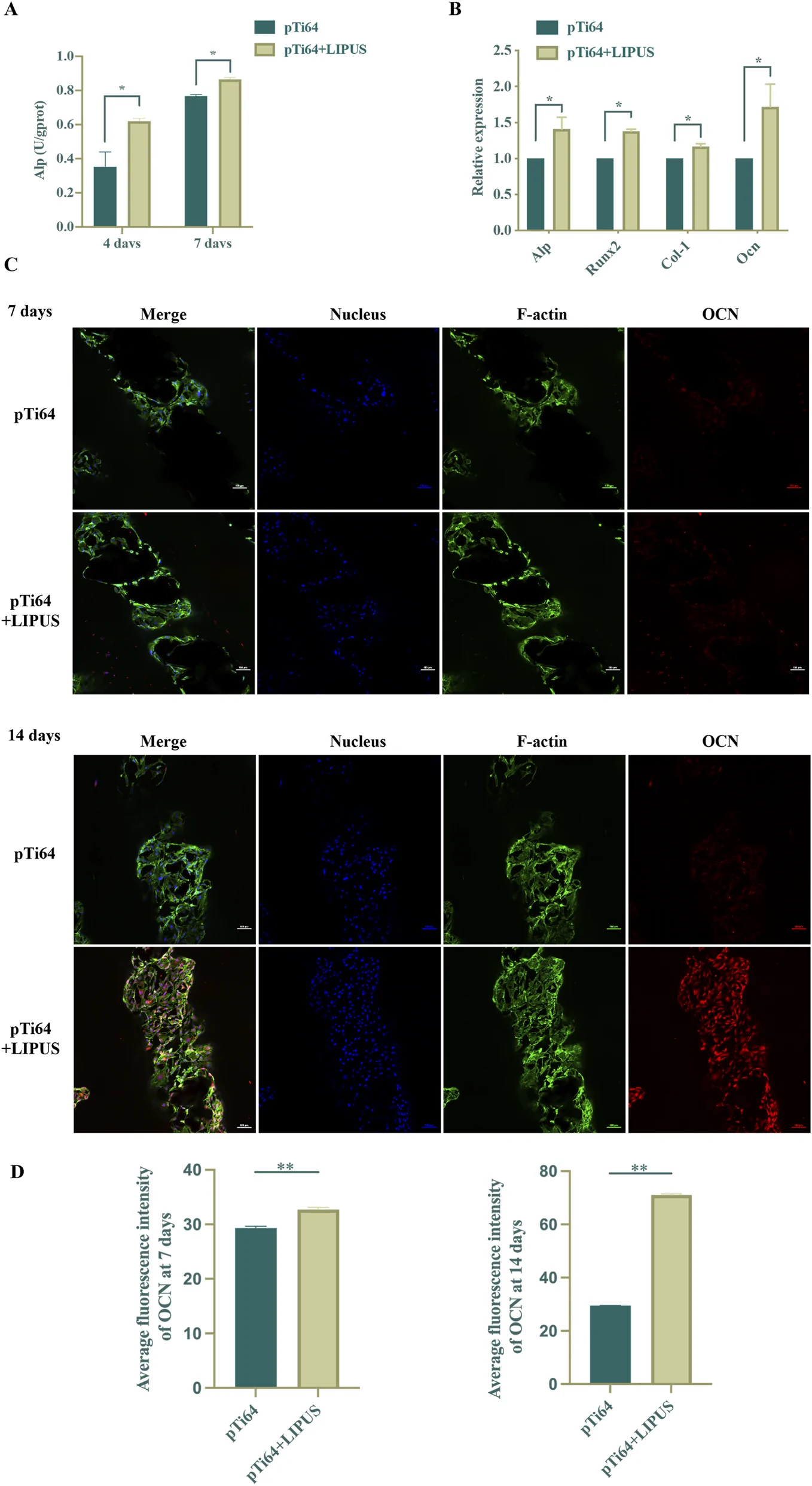
**(A)** ALP activity of MC3T3-E1 cells in pTi64 group and pTi64+LIPUS group at 4 days and 7 days (n = 3, p < 0.05). U/gprot: enzyme activity units (U) per Gram **(G)** of protein (prot). **(B)** Relative mRNA expression of *Alp, Runx2, Col-1* and *Ocn* (n = 3, *p < 0.05). **(C)** Fluorescent staining of MC3T3-E1 cells after incubation for 7 days and 14 days in different groups (red, Texas red for OCN; blue, DAPI for nucleus; green, fluorescein isothiocyanate for F-actin). Scale bar: 100 μm. **(D)** Statistical analysis results of average fluorescence intensity of OCN at 7 and 14 days (n = 3, **p < 0.01).

Real-time quantitative PCR (RT-PCR) was used to quantify the osteogenic differentiation-related genes *Alp*, *Runx2*, *Col-1*, and *Ocn* at 7 days. As Figure 3B illustrates that the mRNA expression levels of *Alp*, *Runx2*, *Col-1*, and *Ocn* were significantly elevated in the pTi64+LIPUS group compared to the pTi64 group.

#### 3.3.2 OCN immunofluorescence staining and imaging

OCN, a low-molecular-weight protein synthesized by osteoblasts, facilitates mineralization and serves as a late differentiation marker. In this study, immunofluorescence staining was used to visualise the distribution of OCN in pTi64 scaffolds after LIPUS stimulation for 7 and 14 days. The immunofluorescence images (Figure 3C) showed that the protein expression of OCN was promoted by LIPUS stimulation. The average fluorescence intensity of OCN was quantified from immunofluorescent images using Image-Pro Plus software (Figure 3D). The results indicate that the pTi64+LIPUS group displayed a higher average OCN level after 7 and 14 days than the pTi64 group.

### 3.4 Metabolite profiling

Intracellular metabolites were extracted and analyzed using by UHPLC-QTRAP-MS/MS after culturing MC3T3-E1 osteoblasts in pTi64 scaffolds with and without LIPUS stimulation for 7 days. Reliable analytical methods and steady detection platforms are indispensable for ensuring high-quality data in terms of metabolomics. Consequently, this study employed PCA, a multivariate pattern-recognition technique. The PCA score plot demonstrated a tight clustering of all QC samples (Figure 4A). The total ion chromatograms extracted from all QC samples demonstrated consistent overlap, similar chromatographic profiles, and stable retention times throughout the process (Figure 4C). The study confirms the stability and reproducibility of UHPLC-QTRAP-MS/MS. Although the PCA scores plot indicated minimal separation between the two groups, subsequent OPLS-DA analysis (Figure 4B) provided further insights was employed to identify key metabolites that significantly differentiated between the pTi64+LIPUS and pTi64 groups. The distinct separation between the two groups indicates that LIPUS treatment significantly altered the metabolism of MC3T3-E1 osteoblasts, despite minimal changes in overall osteoblast metabolism. VIP scores from the first or second principal component, derived via partial least squares, were utilized to identify discriminative features that contribute to group separation in OPLS-DA models. A feature is deemed significant if its VIP value is 1.0 or higher. Significant variables responsible for clear separation were identified using a two-tailed Student’s t-test with P-values less than 0.05 and FC thresholds of ≥ 1.2 or ≤ 0.83. Refer to the volcano plot (Figure 4D) for a visual representation. In Figure 4D, metabolites that were upregulated in the pTi64+LIPUS group compared to the Ti64 group appeared on the right side of the valley, whereas downregulated metabolites were located on the left side. In MC3T3-E1 osteoblasts cultured in pTi64 scaffolds under LIPUS, 86 features were identified as potential metabolite biomarkers, with 39 downregulated and 47 upregulated compared to the sham group. Figure 4E provides a summary of the clearly identified metabolites. The data illustrated in Figure 4E reveal significant metabolic changes in MC3T3-E1 cells within porous scaffolds induced by LIPUS. In the pTi64+LIPUS experimental group, there was an observed increase in the levels of pantothenol, D-pantothenol, 1-aminocyclohexanoic acid, carnitine C22:2, hexanoyl glycine, and suberic acid. Conversely, the levels of (4E,7E,10Z,13E,16E,19E)-docosa-4,7,10,13,16,19-hexanoic acid, 16-dehydroprogesterone, choline, and prostaglandin E2 were found to decrease.

**FIGURE 4:**
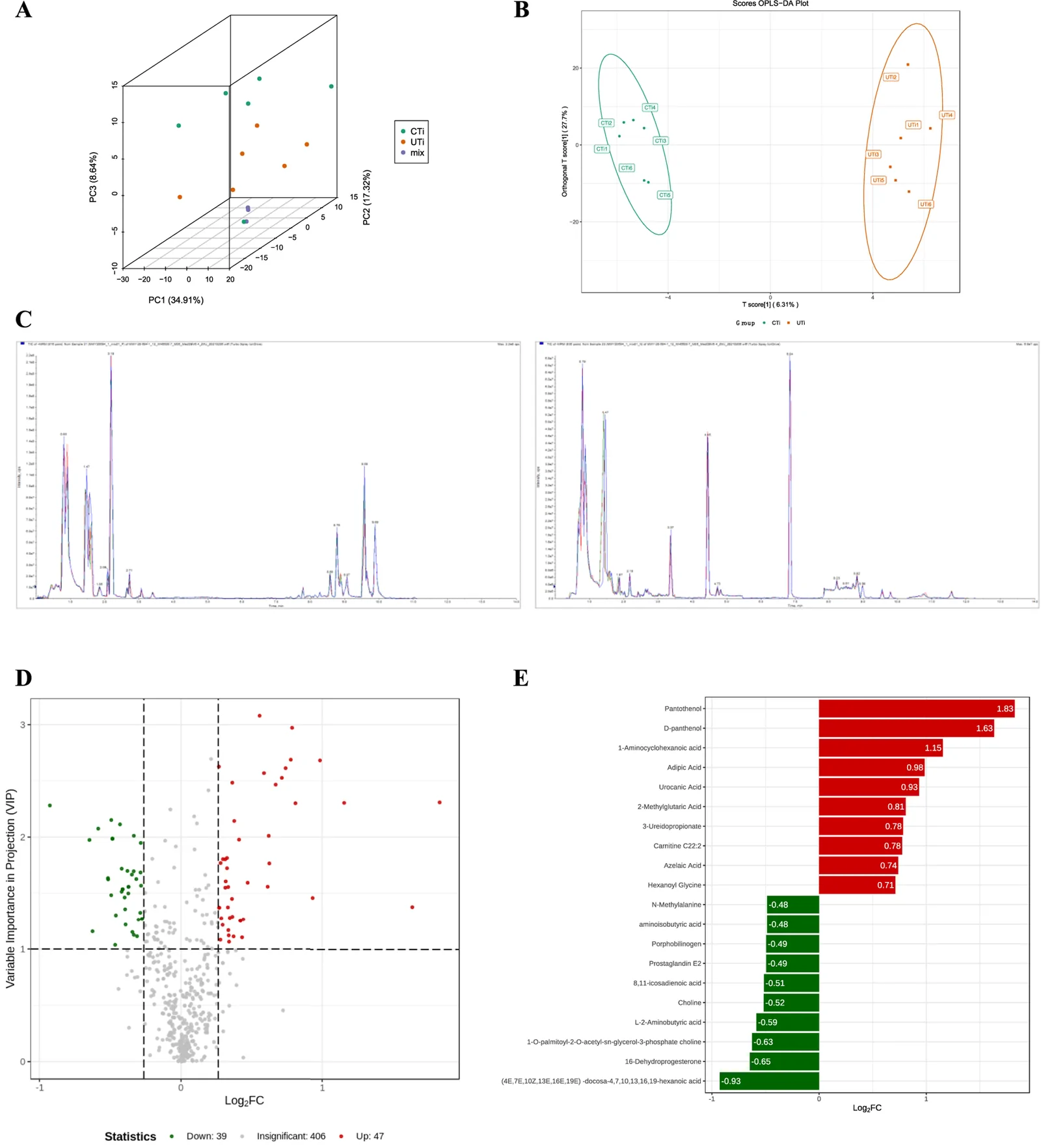
**(A)** Three-dimensional PCA score plots and **(B)** OPLS-DA score plots for metabolome of MC3T3-E1 cells stimulated with (UTi) and without (CTi) LIPUS (n = 6). **(C)** TICs obtained in electrospray ionization (ESI) negative and positive ion mode from all QC samples (n = 10), respectively. ESI (−) conditions show on the left panel; ESI (+) conditions show on the right panel. **(D)** Volcano plot of differential metabolites between CTi and UTi. Red point, green point, and gray point indicate the metabolites that were significantly upregulated, significantly downregulated, and non-significantly different, respectively. **(E)** The bar plot of top 20 upregulated metabolites and downregulated metabolites (VIP > 1, FC > 1.2 or < 0.83, and P < 0.05).

A heat map with hierarchical clustering was employed to enhance data intuitiveness. On the heat map (Figure 5A), clearly illustrates the altered metabolite concentration patterns across samples. The findings indicated that the majority of significantly altered metabolites belonged to the categories of organic acids and their derivatives, amino acids and their metabolites, and fatty acids. Pathway enrichment analysis of identified metabolites was conducted using the KEGG database for visualizing and interpreting metabolic changes at the system level. The fluctuating metabolites were primarily associated with one-carbon pool metabolism via folate, pantothenate and coenzyme A (CoA) biosynthesis, glycerolipid metabolism, and arachidonic acid metabolism (Figure 5B). The altered metabolites were enriched in pathways related to the tricarboxylic acid cycle, amino acids biosynthesis, antifolate resistance, vitamin digestion and absorption, mineral absorption, and folate biosynthesis. LIPUS treatment in pTi64 scaffolds altered multiple metabolic pathways and cellular processes, potentially contributing collectively to bone formation and maturity. We developed a metabolomic regulation network utilizing the KEGG database, to investigate the regulatory interactions among biologically related molecules (Figure 5C).

**FIGURE 5:**
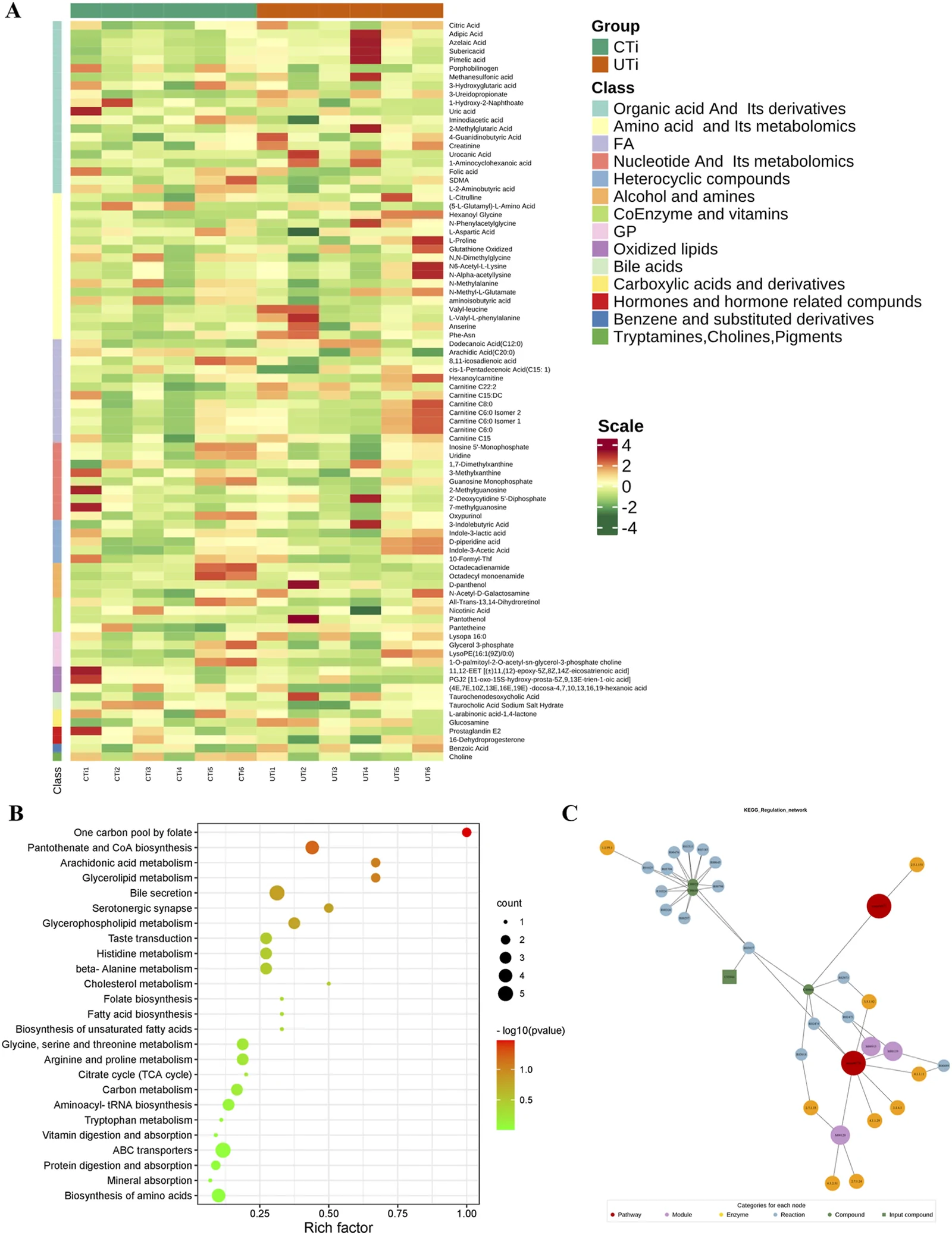
**(A)** Heat map analysis of 86 differential metabolites between MC3T3-E1 cells stimulated with (UTi) and without (CTi) LIPUS. The values of differential metabolites were normalized and shown as a color scale. The high and low metabolite levels were represented as reddish and greenish scales, respectively. **(B)** Statistics of KEGG enrichment. The x-axis indicates the rich factor corresponding to each pathway, and the y-axis indicates name of the KEGG metabolic pathway. The size and color of bubbles represent the number and degree of enrichment of different metabolites, respectively. **(C)** KEGG regulation network. Red point, purple point, yellow point, blue point, green point, and green block indicate pathway, module, enzyme, reaction, compound, and imput compound respectively.

**FIGURE 6:**
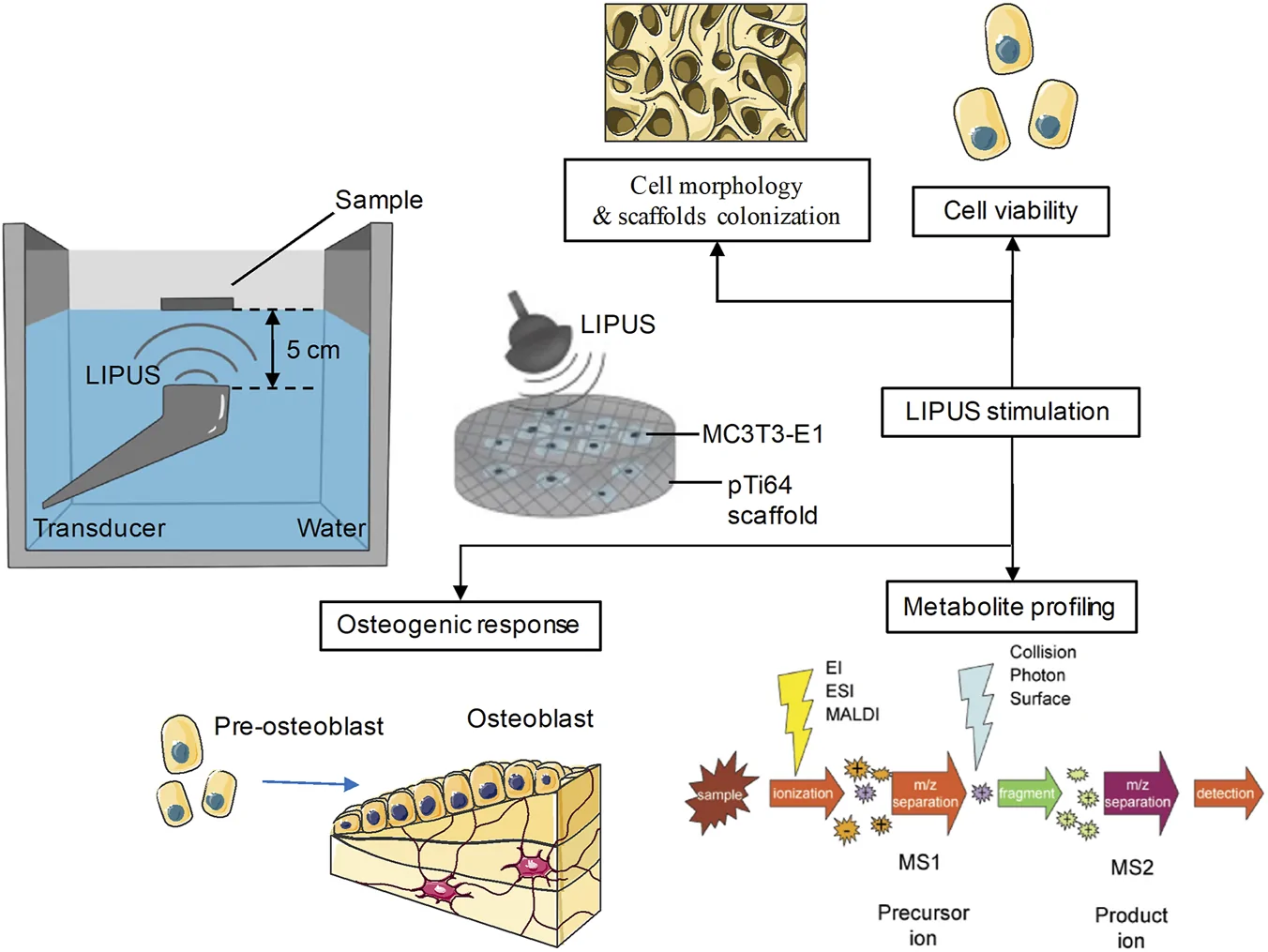
Graphical abstract.

## 4 Discussion

LIPUS enhanced the ingrowth and osteogenic differentiation of MC3T3-E1 cells in pTi64 scaffolds. To investigate the growth and adhesion of osteoblasts in pTi64 scaffolds under LIPUS, we photographed and reconstructed three-dimensional cells in three-dimensional scaffolds using confocal laser scanning microscopy. The results suggest that LIPUS improved cell morphology and scaffold colonisation in the pTi64 scaffolds. We employed a three-dimensional reconstruction technique to observe cell growth in porous scaffolds, offering a more intuitive and vivid perspective compared to other methods. The live/dead cell fluorescent staining and apoptosis assays further indicated that LIPUS reduced cell death and enhanced cell viability. In addition to cell adhesion, activity, and apoptosis, cell differentiation plays a crucial role in bone formation. The activity of ALP was significantly increased by LIPUS treatment. RT-qPCR analysis revealed that mRNA expression of osteoblast-related genes (*Alp*, *Runx2*, *Col-1*, and *Ocn*) were significantly elevated in the pTi64+LIPUS group compared to the pTi64 group. *Alp*, *Cola1*, and *Runx2* are typically expressed in the early phases of osteogenic differentiation, while *Ocn* expression emerges later in the process. Therefore, our results indicate that LIPUS plays an important role in cell adhesion, activity, and differentiation, consistent with previous research findings (Cao et al., 2017).

In our study, we discovered that LIPUS prompts extensive metabolic reprogramming during osteogenic differentiation. A pivotal question emerging from our findings is the mechanism by which a physical stimulus, such as ultrasound, is converted into biochemical signals that subsequently modify cellular metabolism. LIPUS is recognized for its biological effects, which are primarily mediated through micromechanical stress and acoustic radiation force, leading to the activation of a series of mechanotransduction pathways (Zhou et al., 2023). Traditional mechanosensitive pathways involved in the response to LIPUS include the integrin-FAK-MAPK/ERK pathway, calcium influx, and the Hippo pathway effectors YAP/TAZ (Wu et al., 2024). At the metabolic level, YAP/TAZ have been demonstrated to directly regulate amino acid metabolism, encompassing the serine-glycine synthesis pathway and one-carbon unit metabolism (Rachedi et al., 2024). Specifically, YAP/TAZ facilitate glutamine and serine catabolism to support glycine anabolism and modulate key enzymes within the serine-glycine-one-carbon metabolic network (Wu et al., 2017). Based on these observations, we hypothesize that LIPUS, by activating YAP/TAZ mechanotransduction, reprograms cellular metabolism through the upregulation of serine-glycine-one-carbon pathways. This reprogramming potentially enhances nucleotide synthesis, methylation reactions, and redox homeostasis (Sun et al., 2023), which are critical for the rapid proliferation and differentiation of osteoblasts within pTi64 scaffolds.

This hypothesis is strongly supported by our metabolomics data, which indicate that the folate-mediated one-carbon metabolism pathway is one of the most significantly altered pathways in cells treated with LIPUS. Folate-mediated one-carbon metabolism is crucial for the synthesis of purines, thymidylate, and S-adenosylmethionine (SAM), the universal methyl donor involved in DNA, RNA, and protein methylation (Dang et al., 2024). These processes are essential for cell proliferation and lineage specification (Yang et al., 2021). Methylation modifications serve as critical regulators of osteogenic differentiation. Specifically, DNA methylation within the promoter regions of osteogenesis-associated genes, such as *RUNX2* and osterix, modulates their transcriptional activity (Farshdousti Hagh et al., 2015). Concurrently, the methylation of histone marks H3K4me3 influences the chromatin accessibility of loci pertinent to bone-related genes (Yang et al., 2017). We hypothesize that LIPUS may promote osteogenesis by activating YAP/TAZ, reprogramming folate-mediated one-carbon metabolism, and thereby modulating the methylation capacity of osteoblasts. Notably, levels of folate and 10-formyl-tetrahydrofolate were substantially reduced in the pTi64+LIPUS group, suggesting an increased utilization of folate for one-carbon transfer reactions. The KEGG enrichment analysis also identified folate biosynthesis alongside folate-mediated one-carbon metabolism as significantly enriched pathways. These two pathways are functionally interconnected: folate biosynthesis provides the intracellular tetrahydrofolate pool required for one-carbon transfer reactions, while the one-carbon metabolism pathway utilizes these folate cofactors to support nucleotide synthesis and methylation reactions (Joly et al., 2021). In our study, the concurrent enrichment of both pathways likely reflects an increased cellular demand for folate supply and one-carbon utilization during LIPUS-induced osteogenic differentiation, with folate-mediated one-carbon metabolism representing the core functional axis through which LIPUS exerts its metabolic effects. Our findings that LIPUS enhances folate consumption are consistent with previous studies indicating that folate-loaded nanoparticles facilitate osteoblast differentiation and bone regeneration (Santos et al., 2017). Furthermore, our study extends these findings by demonstrating that a physical stimulus may actively accelerate intracellular folate utilization to fulfill the biosynthetic requirements of osteogenic differentiation.

Similarly, LIPUS induced the alteration of pantothenate and CoA biosynthesis in osteoblasts in porous scaffolds. Pantothenic acid, or vitamin B5, is a crucial vitamin B group member and a precursor to CoA, an essential cofactor in fatty acid oxidation (Henriques et al., 2021), the tricarboxylic acid cycle (Sang et al., 2022), and acetyl-CoA production (Karthikaichamy et al., 2021). Increased CoA biosynthesis suggests an enhancement in energy metabolism and could modulate AMPK/mTOR signaling (Miranda-Cervantes et al., 2025), which in turn regulates osteogenic differentiation (Xi et al., 2016; Lin et al., 2026). Our finding is consistent with a previous study by Zhao et al., who reported that pantothenate and CoA biosynthesis are regulated during mesenchymal stem cell osteogenesis induced by astaxanthin (Zhao et al., 2020). Therefore, changes in pantothenate and CoA biosynthesis may contribute to LIPUS-enhanced osteogenic differentiation, at least in part, AMPK/mTOR energy-sensing axis.

Beyond its effects on one-carbon and CoA metabolism, LIPUS significantly restructured amino acid and lipid metabolic pathways. Notable alterations were observed in various amino acid derivatives, such as 5-L-glutamyl-L-amino acid, L-aspartic acid, N,N-dimethylglycine, and N-methylalanine, with pathway enrichment identified in histidine, arginine-proline, alanine-aspartate-glutamate, and cysteine-methionine metabolism. Of particular interest, N,N-dimethylglycine serves as a crucial intermediate within the glycine-serine-one-carbon network (Tibbetts and Appling, 2010), thereby establishing a direct connection between amino acid metabolism and the folate-mediated one-carbon pathway. This linkage implies that LIPUS facilitates a coordinated reprogramming of amino acid and one-carbon metabolism to meet the biosynthetic and epigenetic requirements of osteogenic differentiation. Furthermore, LIPUS led to a reduction in arachidonic acid and glycerol-3-phosphate levels, while modulating glycerolipid and arachidonic acid metabolism. Arachidonic acid has been reported to suppress osteoblast differentiation in MC3T3-E1 cells in a dose-dependent manner (Coetzee et al., 2007; 2009). The reduction in arachidonic acid levels observed in our LIPUS-treated group may therefore contribute to osteogenic differentiation by relieving this inhibitory effect. Concurrent modifications in mineral absorption and vitamin digestion pathways likely indicate the heightened demands for calcium, phosphorus, and vitamins during matrix mineralization. Together, these multi-pathway alterations point to a deliberate and integrated metabolic response rather than random fluctuations.

Our findings align with the existing body of literature on LIPUS-enhanced osteogenesis. However, our study advances this field by offering a comprehensive global metabolomic analysis of how LIPUS influences the intracellular metabolic network of osteoblasts within 3D porous scaffolds. While prior research has predominantly concentrated on phenotypic outcomes or isolated signaling pathways, our data demonstrate that LIPUS triggers a coordinated metabolic reprogramming. This reprogramming involves one-carbon metabolism, CoA biosynthesis, and amino acid/lipid pathways, which are likely orchestrated through YAP/TAZ-mediated mechanotransduction. This integrated perspective bridges the gap between the physical stimulus of ultrasound and the subsequent metabolic processes that ultimately facilitate osteogenic differentiation.

Although our study reveals several important discoveries, certain limitations should be acknowledged. Firstly, we did not directly quantify the acoustic field intensity at the cell layer, though all group comparisons were performed under strictly identical conditions. Secondly, the focal travel range of our confocal microscope system limited our morphological analyses to the upper 850-μm layer of the 3-mm-thick scaffolds, leaving the deeper central region uncharacterized. Thirdly, the mechanistic model involving YAP/TAZ-mediated metabolic reprogramming remains hypothetical, as functional validation experiments were not conducted. Fourthly, our metabolomic analysis was limited to a single time point at Day 7, and we did not apply multiple-testing correction or normalize to total protein/DNA content. These methodological constraints should be considered when interpreting our findings, and they point to clear directions for future investigation.

## 5 Conclusion

In this study, we systematically investigated the effects of LIPUS on the biological behavior and metabolic network of MC3T3-E1 osteoblasts cultured within pTi64 scaffolds. Our findings demonstrate that LIPUS not only enhances cell adhesion, viability, and osteogenic differentiation, but also induces a comprehensive metabolic reprogramming involving folate-mediated one-carbon metabolism, pantothenate and CoA biosynthesis, and amino acid/lipid pathways. These results suggest that LIPUS induces a coordinated metabolic remodeling in osteoblasts within 3D scaffolds. Our findings provide a metabolomics-based perspective on the cellular response to LIPUS and support further exploration of LIPUS-combined 3D-printed titanium alloy scaffolds for large bone defect repair.

## Data Availability

The raw data supporting the conclusions of this article will be made available by the authors, without undue reservation.
